# Art and Disease

**Published:** 1894-02-03

**Authors:** 


### Art and Disease.

### IV.—ARTISTIC DEFORMITY.

The expression "artistic deformity" may be thought a contradiction in terms. True art must exclude, it may be argued, any representation of the abnormal or the imperfect. That artists have commonly thought otherwise is clear from an examination of their works, but it also appears that the study of the unbeautiful has been mainly pursued in periods of degeneration of art. The greatest artists have been sometimes attracted, however, either by the necessities of their subject, or by a keen interest in natural phenomena, to spend their force on depicting distorted aspects of humanity, and the pathological interest of such pictures or statues is great just in proportion to the sacrifices rendered by the artist's perception of beauty to his sense of truth.

Egyptian art delights in the grotesque, and is specially rich in the representations of dwarfs. The Pharoahs seem to have had a special fancy for dwarfs, and their court was not considered complete without one. The gods Phthah and Bes were always represented of minute stature, and two well marked types have been preserved by the artists in depicting them, betraying by accurate traits numerous characteristics of this class of deformity in the present day. In the one case all the parts are in proportion, and no trace of disease is present; in the other every symptom of rickets is conveyed, in the long body, swollen head, short, massive limbs, and the peculiar curve of the legs.

The Greeks have left scarcely a trace behind of morbid interest in physical imperfection. The one important exception is a bust, presumably of Æsop, preserved at the Villa Albani in Rome, in which has been recognised a most accurate and scientific delineation of that variety of spinal curvature known as cyphosis.

From the time of the Renaissance the figures of dwarfs and fools are continually introduced into every kind of picture. Their portraits were painted by the great masters, and every description of deformed stature and arrested development has been accurately rendered. They hold a less honourable place also as models for the grotesque heads which were so extensively used in ornamentation, and, grossly exaggerated as many of these are, it is easy to recognise the special symptoms of the nervous spasm or permanent contortion which the artist must have had under his observation. Nowhere is the physician more directly opposed to the artist in his judgment than on the subject of these grotesques. Take for example the grotesque head carved on the base of the tower of Santa Maria Formosa at Venice. Of this Mr. Ruskin says:* "A head—huge, inhuman, and monstrous—leering in bestial degradation, too foul to be either pictured or described, or to be beheld for more than an instant. Yet let it be endured for that instant, for in that head is embodied the type of the evil spirit to which Venice was abandoned in the fourth period of her decline. . . . . This head is one of the many hundreds which disgrace the latest buildings of the city, all more or less agreeing in their expression of sneering mockery, in most cases enhanced by thrusting out the tongue. They are evidences of a delight in the contemplation of bestial vice and the expression of low sarcasm, which is, I believe, the most hopeless state into which the human mind can fall." It is rather refreshing, after this uncomfortable judgment on poor Venice, to turn to the doctor's diagnosis. M. Charcot writes: † "In our opinion this distortion of the features, which gives the mask so grotesque and hideous an aspect, is by no means the result of a mere artistic whim. Nature, in her infinite variety of forms, is a mine of inexhaustible richness, where the beautiful and the ugly, the harmonious and the deformed are mingled with intermediary degrees. The artist of Santa Maria Formosa, seeking for a grotesque type, seems to us to have met one in his path, to have seen it with his eyes, seized it in passing, and reproduced it with a fidelity which enables us at this day to recognise in it the signs of a pathological deformation, of a nervous affection, clearly defined, of which we have had under our notice at La Salpêtrière very interesting examples. The case in question is evidently a spasm of the face, of a special nature, existing often in hysterical males or females in conjunction with hemiparalysis of the limbs, and presenting characters so marked that it is impossible to confound it with any other facial and spasmodic affection. The spasm is localised to one half of the face. It invades the eye, which is partly or wholly closed; it draws the nose on one side, raising one nostril; and invades also the lower part of the face, both chin and lips being violently drawn aside. Finally, the protruding tongue is crooked, the point being thrust to the side where the spasm exists, and with an exaggeration which constitutes in itself one of the most characteristic traits of the affection." Thus the physician comes to the rescue of the artist, and reveals a mere naturalistic tour-de-force in what the artist has read as "a delight in the contemplation of bestial vice."

Among the most celebrated portraits of dwarfs is the one by Mantegna in the "Triumph of Julius Cæsar," at Hampton Court, where the face shows unmistakeably the combined signs of scrofula and rickets. Leonardo da Vinci has left in a collection of grotesque drawings a sketch, evidently drawn from life, of a dolicocephalous crétin with goitre. Paul Veronese has left several examples of dwarfs, the most clearly marked type being one in the Fainting of Esther, where the malformation of the head suggests hydrocephalus.

The Spanish school has many examples of dwarfs and idiots, and Velasquez especially seems to have had a marked fancy for their peculiarities. No less than seven pictures devoted to this subject are preserved in the Prado Museum at Madrid, by him. It is unnecessary to recall the various points of realism which the experienced eye can detect. In the Dutch school they are almost equally prominent, and it is conclusively evident that the best artists, far from being repelled by physical or mental peculiarities, have frequently spent their best energies on handing them down to posterity.

* Stones of Venice, vol. III., p. 121.

† Les Difformes et les Malades dans l'Art. Par J. M. Charcot et Paul Richer.

